# *KCNH2* p.Gly262AlafsTer98: A New Threatening Variant Associated with Long QT Syndrome in a Spanish Cohort

**DOI:** 10.3390/life12040556

**Published:** 2022-04-08

**Authors:** Rebeca Lorca, Alejandro Junco-Vicente, Alicia Pérez-Pérez, Isaac Pascual, Yvan Rafael Persia-Paulino, Francisco González-Urbistondo, Elías Cuesta-Llavona, Bárbara C. Fernández-Barrio, César Morís, José Manuel Rubín, Eliecer Coto, Juan Gómez, José Julián Rodríguez Reguero

**Affiliations:** 1 Unidad de Referencia de Cardiopatías Familiares-HUCA, Área del Corazón y Departamento de Genética Molecular, Hospital Universitario Central de Asturias, 33011 Oviedo, Spain; lorcarebeca@gmail.com (R.L.); uo218576@uniovi.es (E.C.-L.); cesar.moris@sespa.es (C.M.); josemanuel.rubin@sespa.es (J.M.R.); eliecer.coto@sespa.es (E.C.); juan.gomezde@sespa.es (J.G.); josejucasa@yahoo.es (J.J.R.R.); 2 Heart Area, Hospital Universitario Central de Asturias, 33011 Oviedo, Spain; ajuncovicente@gmail.com (A.J.-V.); yvanrafael.persia@sespa.es (Y.R.P.-P.); frangonzalez_95@hotmail.com (F.G.-U.); 3 Instituto de Investigación Sanitaria del Principado de Asturias (ISPA), 33011 Oviedo, Spain; 4 Pediatric Area, Hospital Universitario Central de Asturias, 33011 Oviedo, Spain; alicia.perez@sespa.es (A.P.-P.); barbaracovadonga.fernandez@sespa.es (B.C.F.-B.); 5 CIBER-Enfermedades Respiratorias, 28029 Madrid, Spain

**Keywords:** long QT syndrome, *KCNH2* gene, inheritable arrhythmogenic disorder, genetic testing

## Abstract

Long QT syndrome (LQTS) is an inherited (autosomal dominant) channelopathy associated with susceptibility to ventricular arrhythmias due to malfunction of ion channels in cardiomyocytes, that could lead to sudden death (SD). Most pathogenic variants are in the main 3 genes: *KCNQ1 (LQT1)*, *KCNH2 (LQT2)* and *SCN5A (LQT3)*. Efforts to improve the understanding of the genotype-phenotype relationship are essential to improve the medical clinical practice. In this study, we identified all index patients referred for NGS genetic sequencing due to LQTS, in a Spanish cohort, who were carriers of a new pathogenic variant (*KCNH2* p.Gly262AlafsTer98). Genetic and clinical family screening was performed in order to describe its phenotypic characteristics. We identified 22 relatives of Romani ethnicity, who were carriers of the variant. Penetrance reached a 100% and adherence to medical treatment was low. There was a high rate of clinical events, particularly arrhythmic events and SD (1 in every 4 patients presented syncope, 1 presented an aborted SD, 2 obligated carriers suffered SD before the age of 40 and 4 out of 6 carriers of an implantable cardioverter-defibrillator (ICD) had appropriate ICD therapies. Correct adherence to medical treatment in all carriers should be specially encouraged in this population. ICD implantation decision in non-compliant patients, and refusing left cardiac sympathetic denervation, should be carefully outweighed.

## 1. Introduction

Long QT syndrome (LQTS) is an inherited arrhythmogenic disorder (channelopathy), characterized by prolonged ventricular repolarization (QTc interval) associated with an increased susceptibility to life-threatening arrhythmic events (LAE), such us torsade de pointes and polymorphic ventricular tachycardia [1,2,3]. In contrast to other less common channelopathies, the prevalence of LQTS is at least 1 in 2.500 caucasian live births [4]. The pathogenic basis of this hereditary disease involves alterations in the functioning of the ion channels of the cardiomyocytes [1,2]. Although more than 17 related genes have been described, most pathogenic variants are identified in the three principal genes: *KCNQ1, KCNH2*, and *SCN5A*; causing LQT1, LQT2, and LQT3, respectively [1,5]. Therefore, LQT1 is due to pathogenic variants in *KCNQ1* gene. This gene encodes the subunit KV7.1 of the voltage-gated potassium channel that is responsible for the outward potassium current *IKs* [6]. Conversely, pathogenic variants in *SCN5A,* encoding the subunit-α NaV1.5 of the voltage-gated sodium channel (responsible for the inward sodium current *INa*), are associated with LQT3 [7]. On the other hand, LQT2 is related to the *KCNH2* gene, also named (hERG, human ether-a-go-go related gene). This gene encodes the pore-forming α-subunit of potasium channel (Kv11.1), responsible for the inward rectifying potassium current [8]. Therefore, *KCNH2* expression plays an essential role in the final repolarization of the ventricular action potential [9]. The gene contains 16 exons on locus chromosome 7q36.1. The subunit protein is an important part of the channel that conduct the rapidly activating delayed rectifier K+ current (IKr) in the cardiomyocyte sarcolemma membrane [9,10].

Stratification of the arrhythmic risk of each patient with LQTS is essential. Many research studies have tried to analyze genotype-phenotype relations based on cohorts of index cases carriers of pathogenic variants in the same gene [1,11]. However, in this channelopathy, incomplete penetrance and variable expressiveness is a common characteristic [12]. Therefore, it is necessary to continue with the investigation and the description of clinical manifestations in large cohorts including not only index cases, but also large affected families. This would help to establish strength genotype-phenotype and arrhythmic risk relationships. In this context, we aimed to describe a new genetic variant on *KCNH2* gene associated with LQT2 in a Spanish cohort, analyzing penetrance, arrhythmic risk, and clinical presentation.

## 2. Materials and Methods

### 2.1. Study Population

In this retrospective clinical study, we reviewed all consecutive index cases referred for genetic testing with LQTS diagnosis. We identified all index patients carriers of the *KCNH2* p.Gly262AlafsTer98 (c.785delG: NM_000238) variant (Supplementary Figure S1). Three index cases, carriers of this pathogenic new variant responsible for LQT2, were identified and included in the study. Clinical and genetic screening in all available relatives of these 3 index patients was systematically performed. All patients who wished to participate, signed written consent to grant access to their genetic data for investigational purposes and the research protocol followed institutional ethics guidelines. This study was evaluated by the local Ethical Committee (CEImPA 2021.528).

Clinical data and demographic information were investigated, recording personal and family history of symptoms, arrhythmic events, electrocardiographic parameters, devices implantations and therapies. Echocardiogram was not systematically performed in relatives screening, as LQTS is a channelopathy. Implantable cardioverter defibrillators (ICD) registries were also reviewed.

We established positive phenotype according to the QTc of the ECGs of the individuals studied, understood as a prolonged QTc interval (QTc longer than 440 milliseconds for men and QTc longer than 460 milliseconds for women, measured in lead V5, and corrected by Bazett’s formula) [13,14]. For the penetrance calculation, we excluded five patients who had suffered sudden death without available ECG for review. In addition, on the one hand, there was an asymptomatic patient with positive phenotype (prolonged QTc) who refused to perform genetic study. On the other hand, 2 carriers had to be excluded for penetrance calculation (an obligate carrier who refused clinical evaluation and a positive carrier who presenting a non-evaluable phenotype due to his too young paediatric age).

### 2.2. Genetic Testing

Blood samples were obtained from all patients who accepted to undergo genetic testing, collected in a 9 mL tube with EDTA anticoagulant. We isolated DNA from their peripheral blood leukocytes by standard salting-out method, a simple and non-toxic DNA extraction technique that isolates a high-quality DNA from the whole blood [15].

Genetic testing was carried out form DNA samples from all referred patients. 

On the one hand, LQTS index patients were sequenced by NGS (NGS next-generation sequencing) with a total of 210 genes that have been associated with cardiovascular disease (Supplementary Table S1), including LQTS-associated gene. These genes were sequenced with the Ion Torrent technology that uses semiconductor chips and the Ion GeneStudio S5 Sequencer (Termo Fisher Scientific). The detailed procedure was previously reported [16,17].

The raw data was processed with the Torrent Suite v5 software. Reads assembling and variant identification were performed with the Variant Caller (VC). Ion Reporter (Thermo Fisher Scientific, Waltham, MA, USA), and HD Genome One (DREAMgenics S.L., Oviedo, Spain) softwares were used for variant annotation, including population, functional, disease-related and in silico predictive algorithms. The Integrative Genome Viewer (IGV, Broad Institute, Cambridge, MA, USA) was used for the analysis of depth coverage, sequence quality, and variant identification.

On the other hand, familial screening for *KCNH2* p.Gly262AlafsTer98 variant was performed by sanger sequencing in an ABI3130XL sequencer (Thermo Fisher Scientific, Waltham, MA, USA). Interpretation of all gene variants with an allele frequency < 0.01 was based on the American College of Medical Genetics and Genomics (ACMG-AMP) 2015 Standards and Guidelines [18]. 

Only those patients carriers of *KCNH2* p.Gly262AlafsTer98 variant (index or relatives) were included in this study. 

### 2.3. Statistical Analysis

Statistical analyses were performed with SPSS v.25 (SPSS Inc., Chicago, IL, USA). Descriptive data for continuous variables are presented as mean + SD and as frequencies or percentages for categorical variables. The chi-square test or Fisher exact test were used to compare frequencies. *p* < 0.05 was considered to be significant.

## 3. Results

From all consecutive index patients referred for genetic testing for LQTS suspicion (227 patients), we identified 3 index patients with LQTS carriers of the pathogenic variant *KCNH2* p.Gly262AlafsTer98. This variant is not registered in the databases or in the literature. Clinical characteristics are summarized in Table 1. 

**Figure 1:**
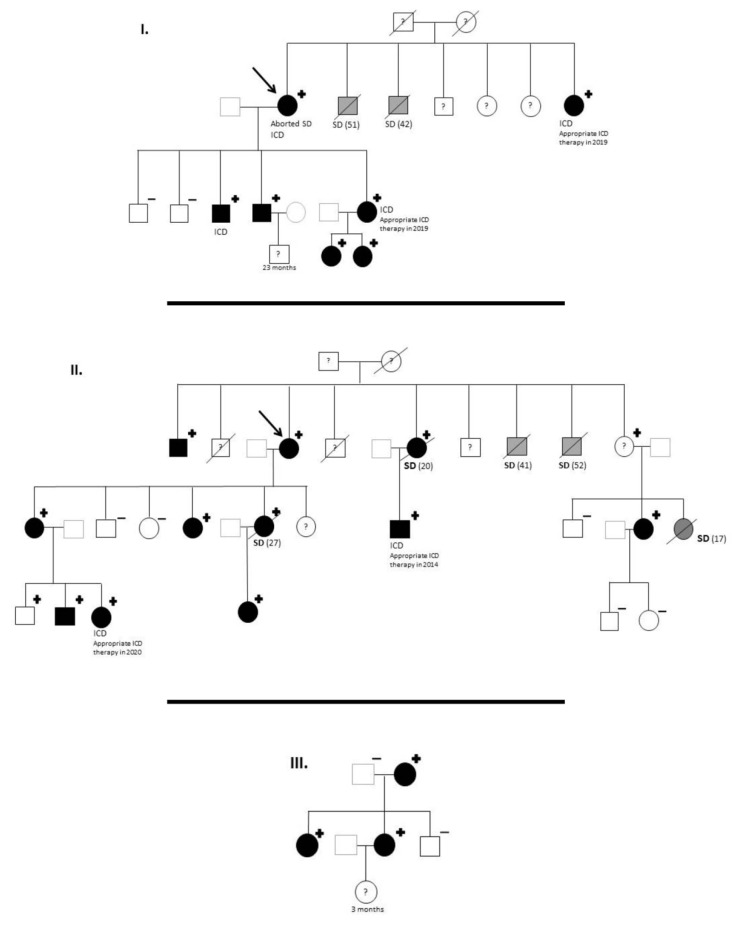
*KCNH2* p.Gly262AlafsTer98 carriers: Family pedigrees. ICD, implantable cardioverter-defibrillator; SD, sudden death. Age of deceased in patients due to SD, in brackets. Symbols denote sex and disease status: “+”, carriers; “−”, noncarriers; without sign, genetic status unknown; box, male; circle, female; black darkened, long QT syndrome phenotype (prolonged QTc in electrocardiogram); grey darkened, unexplained SD; symbol clear, negative phenotype (normal QTc); “?”, unknown phenotype; slashed, deceased; arrow, proband.

Two of the index patients belong to a long-related Spanish family of Romani ethnicity. The third index patient was also of Romani ethnicity. However, we were unable to identify the familial pedigree connection with the previous 2. Family pedigree is shown in Figure 1. 

Within these 3 families, there were 7 (4 men and 3 women) sudden deaths (SD) in previously healthy people and of non-accidental causes. One of them was a woman with aborted sudden cardiac death (SCD) due to ventricular fibrillation (VF). Two other woman, who suffered SD at rest, were obligated carriers. Therefore, positive LQTS phenotype was assumed. 

The mean age of the patients who died from SD was 35.6 ± 13.1 years old. All of them occurred prior to their family’s contact with the cardiology department. Therefore, neither genetic evaluation nor ECG were available. During the follow-up and to date, no other relatives have suffered any other fatal outcomes.

The women who suffered an aborted SCD was the index patient of Family 1 (Figure 1). She suffered VF at age of 24 and was successfully resuscitated with no sequelae. Her ECG showed a prolonged QTc interval and genetic testing identified the nonsense variant *KCNH2* p.Gly262AlafsTer98.

After family screening, a total of 22 *KCNH2* p.Gly262AlafsTer98 carriers from these 3 families were identified, thanks to cascade genetic testing (including 3 obligated carriers). Genetic analysis was not performed in children younger than 3 years old, according to their parents’ wishes. Moreover, 1 patient (with positive phenotype) refused to undergo genetic study. Consanguinity between the positives was not present. Men represented only the 23% of carriers, whereas 77% were woman. The mean age of the carriers was 23.7 ± 13.9 years old, being the youngest a 4-year-old boy.

During family screening, all patients underwent clinical evaluation with ECG before genetic testing. All non-carriers had a normal QTc on the ECG and did not present cardiological symptoms related to channelopathies.

**Figure 2:**
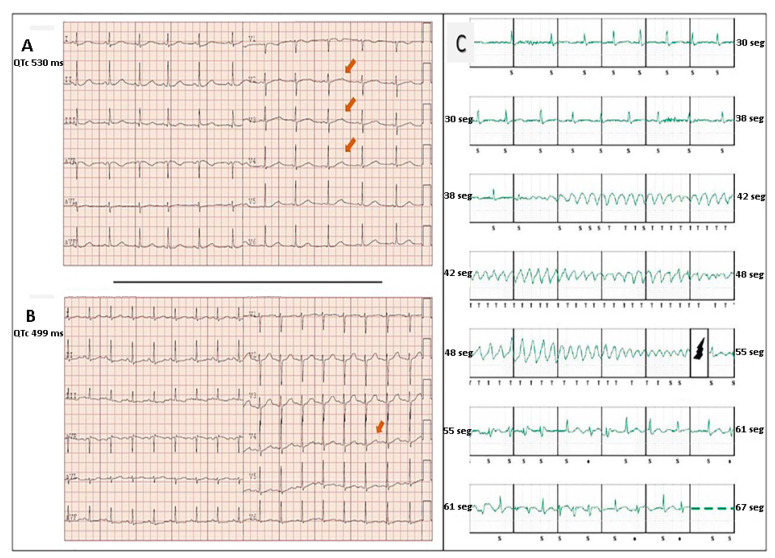
Two ECGs. (**A**) 17-year-old woman with long QT syndrome, a carrier of the *KCNH2* variant. The ECG in sinus rhythm shows jagged T waves in typical precordial leads of LQTS type 2 (arrows) with a QTc of 530 ms. (**B**) ECG of his mother, also a carrier of the variant, and in turn, a carrier of ICD in secondary prevention. Sinus rhythm with jagged T waves and prolonged QTc is also seen (arrow). (**C**) Sudden aborted death of the mother. Subcutaneous ICD registry where appropriate therapy can be observed. Spontaneous torsade de pointes that is detected and effectively defibrillated by the device.

Penetrance was calculated as the proportion of individuals carrying the *KNCH2* pathogenic (positive genotype for LQTS) that also expressed the associated trait (positive phenotype). Phenotype was considered positive if the carrier exhibited long QT in the ECG or an obligated carrier had suffered sudden death at young age without prior clinical evaluation (2 obligated carriers women (<30 years old) who died suddenly were assumed to had a positive phenotype). A child under 5 years old was excluded for penetrance evaluation and information about one carrier is unavailable (patients’ rejection). Therefore, only 20 carriers out of 22 would be considered for clinical penetrance evaluation with a resulting general penetrance of a 100% (Figure 1, Table 1). ECG was only evaluated 18 patients. Thus, 100% of the carriers of the variant with ECG available (18/18) present alterations in the QT interval in the ECG compatible with the diagnosis of long QT syndrome (Figure 2A,B). Mean QTc interval among patients was 520 ± 85 ms.

Nearly 1 in every 4 patients with LQTS in the cohort had presented syncope episodes (23%). Moreover, 3 patients presented cardiac arrest as the first clinical manifestation. None of them had been diagnosed of “possible seizures.” No other cardiac symptoms were recorded in our cohort. Altogether, more than 1 in every 3 LQTS patients (36%), carriers of this pathogenic variant, have had symptoms in their youth (mean age of onset 22.6 ± 10.6 years old)

All positive genotype patients, including paediatric patients, were advised to undergo beta-blocker therapy from the moment of diagnosis. Six patients (27% of patients with positive genotype, 2 men and 4 women) underwent ICD implantation. The first implantation was performed at a mean age of 22 ± 10.7 years old. Three of them were performed in primary prevention (due to syncope) and the other 3 in secondary prevention (aborted SD due to VF and 2 presented syncope with ventricular tachycardia registered in subcutaneous holter). During follow-up, 4 of the 6 patients had an appropriate discharge from the device due to a malignant tachyarrhythmia. One took place, during the puerperium, in a 22 years-old woman that had interrupted beta-blocker, on her own, despite medical advice. The second one occurred in a 19 years-old man who suffered VF properly aborted by the ICD. The last two occurred in a 45 years-old and 22 years-old women whose ICD properly treated both torsade de Pointes episodes (Figure 2C).

Overall, the presence of malignant events among this LQTS cohort is very notable. If we considered that there were other 5 sudden deaths that are suspected carriers (Figure 1), it could be said that life-threatening events appeared in nearly half of the cases (44%, 12/27).

We did not find significant differences between genders, neither in penetrance (as it is 100% in both genders) nor in life-threatening cardiological events. Duration of QTc showed no statistical differences between genders either.

## 4. Discussion

Performing a high standard individualized and precision medicine can be considered the actual challenge for clinical practice. In this scenario, current knowledge of genetics in medicine is helping to materialize this clinical dream. This is particularly notable in inherited diseases, such as channelopathies, including LQTS [19].

Despite the fact that the main genes related to LQTS have been identified many years ago, strong relationships between the specific variant, its phenotype, and its arrhythmic risk are, unfortunately, still missing.

Loss-of-function variants in *KCNH2* gene can alter potassium channel function, leading to an increase of the ventricular action potential duration. This can be considered the pathophysiological basis of LQTS [1,20]. More than half of the pathogenic variants described on the *KCNH2* gene are nonsense [10,21]. Thus, it appears that most LQT2-linked variants likely decrease the IKr repolarization current by decreasing the synthesis of the Kv11.1 channel.

Individual risk stratification is essential. Classically, the duration of the QTc was considered the strongest predictor [22]. However, there is no strict correlation of its duration with clinical events, and it is known that clinical phenotypes are different depending on the affected gene and/or concrete variant [1,23]. Therefore, numerous studies and consensuses documents also have highlighted the importance of the specific genotype in the arrhythmic risk prediction [1,2,24,25,26,27]. In general, patients with LQT2 and LQT3 are considered to be a greater risk of life-threating events than LQT1 patients. Nonetheless, there are still significant limitations to categorize arrhythmic risk according to specific genetic variant in each gene. Many papers have tried to investigate genotype-phenotype relations based on cohorts of index patients, carriers of pathogenic variants in the same gene [11,23,28]. However, this channelopathy is characterized by incomplete penetrance and variable expressiveness [1,12]. Therefore, establishing strong genotype-phenotype arrhythmic risk relationships, based in index cases, can be challenging. Hence, long series describing long affected families carrying the sane pathogenic variant can be useful to understand this crucial aspect.

A-subunit of *KCNH2* protein form a tetramer that inserts into the cell membrane to form the functional potassium channel. Each subunit is composed by 6 α-helical transmembrane segments (S1 to S6), where the K+-selective pore is found between S5 and S6. The transmembrane region of the *KCNH2* encoded channel was defined as the coding sequence involving amino acid residues from 398 through 657, being transmembrane pore-loop region (S5-loop-S6 region) from 552 to 657, with the N-terminus region defined before residue 398, and the C-terminus region after residue 657 [28,29]. Variants that affect the pore-loop region have been described as more arrhythmogenic and pathological [29,30]. Moreover, it has been described that patients with non-missense mutations, mainly frameshift/nonsense mutations were at significantly higher arrhythmogenic risk than those with missense mutations [28].

In *KCNH2* p.Gly262AlafsTer98 variant, the sequence change creates a premature translational stop signal in the *KCNH2* gene, causing for LQT2. The change takes place in the N-terminus region, in the Serine located at the 261 position and it is expected to result in an absent or disrupted protein. To the best of our knowledge, this variant has not been previously described in the scientific literature nor in NHLBI Exome Sequencing, 1000G projects, or GnomAD o ClinVar databases. However, other pathogenic *KNCH2* nonsense variants in close positions have been described, such as *KCNH2* p.Glu229Te [5,28], *KCNH2* p.Thr152fs [31] or *KCNH2* p.Leu380fs [5,32].

LQTS is an autosomal dominant cardiac inherited condition that can present with syncope, palpitations associated with premature ventricular beats or sudden cardiac death. Although clinical manifestations seem to be less frequent in pediatric age than in youth, all children with a positive phenotype are recommended treatment with a beta-blocker [1,24,27]. Despite possible variability in clinical penetrance, in our cohort, all *KCNH2* p.Gly262AlafsTer98 variant carriers clinically evaluated presented positive phenotype. Moreover, the early appearance of symptoms and high arrhythmic risk within this families is striking, especially in the youth. Despite the pathogenic variant is not located in the pore-loop region, the arrhythmic risk in both genders is high. In fact, 3 patients suffered fatal SCD before the age of 30, and another 4 patients around this age presented aborted life-threating malignant arrythmias. In LQT2, characteristically, arrhythmic events have been described related to states of rest/sleep and emotions, unlike LQT1, and being rare during exercise [1,3]. In this sense, within this cohort, all SD that happened prior to familial evaluation occurred during rest or sleep. Moreover, the aborted SCD assisted by the emergency services, also occurred during rest.

An increased risk of arrhythmias in the postpartum period has also been described in affected women [33,34,35,36]. In fact, the families presented in this study, 2 malignant events occurred during this period: a 27-year-old woman suffered sudden death after giving birth, and another 22-year-old woman suffered an aborted polymorphic tachycardia (by the ICD) during puerperium.

Nowadays, betablockers treatment for LQTS remains the cornerstone of medical treatment. Nonselective beta-blockers and especially propanolol and nadolol are considered first-line therapy, having shown a marked reduction in the risk of malignant events (syncope, aborted or fatal SCD) over time and in all age ranges [37]. According to current guidelines, device therapy should be reserved for those symptomatic patients despite β-Blockers therapy [24,27]. Moreover, there are also other effective therapies to consider before ICD implant such as other medications, like mexiletine, that has been shown to be useful to shorten the QT interval [38,39], and left cardiac sympathetic denervation (LCSD) [40,41]. LCSD only requires a minimally invasive short intervention. In carefully selected patients, LSCD and beta-blockers may be appropriate alternatives to avoid ICD implantation in primary prevention [24,26], that still allows ICD implantation if necessary.

In our cohort, adherence to therapy was, in general terms, suspected to be very low. Low therapy adherence may explain not only the high rates of life-threating events, but also the high percentage of ICD implants. In fact, the 22 years-old woman who had an appropriate discharge from the ICD during the puerperium, admitted having interrupted beta-blocker therapy. However, ICD implantation should be carefully outweighed, as the incidence rate of complications following the ICD implant, especially in young patients is not neglectable [42]. Therefore, an extra effort in clinical management of this specific LQTS patients of Romani ethnicity should be invested. Improving adherence to treatment and considering other treatments, like LCSD, is of utmost importance.

We found some limitations. The management of our cohort was conditioned by the low medical treatment adherence and refusal to underwent LSCD, when offered. Information about genotype and/or phenotype about some relatives was impossible to obtain (due, in general, to the rejection of some individuals). SD occurred without neither genotypic nor phenotypic confirmation in 7 patients (2 obligated carriers and the other 5 suspected carriers but without confirmation). Moreover, the description of this *KCNH2* variant’ phenotype, and is not applicable to other variants in this gene. Furthermore, other factors that could modify the expression of the phenotype, such as socioeconomic status, environment, or lifestyle, could not be deeply analyzed.

## 5. Conclusions

*KCNH2* p.Gly262AlafsTer98 is a new pathogenic variant identified in a large Spanish family from Romani ethnicity. If other carriers were identified, caution should be taken in family evaluation, due to its high penetrance (100%) and high frequency of life-threatening arrhythmic events. Correct adherence to medical treatment in all carriers should be specially encouraged in this population, particularly during pregnancy and puerperium. ICD implantation decision in non-compliant patients should be carefully outweighed.

## Figures and Tables

**Table 1:** Clinical and genetical evaluation of the affected families.

*KCNH2* p.Gly262AlafsTer98	Family 1	Family 2	Family 3	Total	Relative Percentage in Carriers
Members	11	21	5	37	-
Genetic study	9	14	5	28	-
Genotype +	7	12 *	3	22	-
Phenotype +	7	10	3	20	100% (20/20) **
SD in genotype +	2	5	0	7	32% (7/22)
SD With Unknown Genotype	2	3	0	5	18% (5/27)
Middle age sudden death (years)	46.5	31.2	-	35.6	35.6
Mean QTc of carriers (ms) with availalbe ECG	518	522	481	520	
Symptoms	4	2	0	6	27% (6/22)
Aborted Sudden Death	1	0	0	1	4.5% (1/22)
ICD carriers	4	2	0	6	27% (6/22)
Appropriate ICD Therapies among ICD carriers	2	2	0	4	67% (4/6)
SD plus appropriate ICD therapies among carriers	3	4	0	7	32% (7/22)
SD or appropriate ICD therapies among carriers plus suspected carriers	5	7	0	12	44% (12/27)

SD: Sudden Death; ICD: implantable cardioverter-defibrillator. * Including 3 obligate carriers. ** A child under 5 years old was excluded for penetrance evaluation and information about one obligated carrier is unavailable (patients rejection). Phenotype was considered positive if the carrier exhibited long QT in the ECG (18/18 evaluated patients) or an obligated carrier had suffered sudden death at young age (2 patients < 30 years old).

## Data Availability

Not applicable.

## Supplementary Materials

The following supporting information can be downloaded at: https://www.mdpi.com/article/10.3390/life12040556/s1. Table S1: Genes included in the NGS, including LQTS-associated genes. Figure S1: Sanger electropherogram of KCNH2 exon 4 PCR fragment displaying p.(Gly262AlafsTer98) variant.

## References

[1] Schwartz P.J., Ackerman M.J., Antzelevitch C., Bezzina C.R., Borggrefe M., Cuneo B.F., Wilde A.A.M. (2020). Inherited cardiac arrhythmias. Nat. Rev. Dis. Primer.

[2] Mazzanti A., Maragna R., Vacanti G., Monteforte N., Bloise R., Marino M., Braghieri L., Gambelli P., Memmi M., Pagan E. (2018). Interplay Between Genetic Substrate, QTc Duration, and Arrhythmia Risk in Patients With Long QT Syndrome. J. Am. Coll. Cardiol..

[3] Neira V., Enriquez A., Simpson C., Baranchuk A. (2019). Update on long QT syndrome. J. Cardiovasc. Electrophysiol..

[4] Schwartz P.J., Stramba-Badiale M., Crotti L., Pedrazzini M., Besana A., Bosi G., Gabbarini F., Goulene K., Insolia R., Mannarino S. (2009). Prevalence of the congenital long-QT syndrome. Circulation.

[5] Kapplinger J.D., Tester D.J., Salisbury B.A., Carr J.L., Harris-Kerr C., Pollevick G.D., Wilde A.A.M., Ackerman M.J. (2009). Spectrum and prevalence of mutations from the first 2500 consecutive unrelated patients referred for the FAMILION long QT syndrome genetic test. Heart Rhythm.

[6] Wang Q., Curran M.E., Splawski I., Burn T.C., Millholland J.M., VanRaay T.J., Shen J., Timothy K.W., Vincent G.M., de Jager T. (1996). Positional cloning of a novel potassium channel gene: KVLQT1 mutations cause cardiac arrhythmias. Nat. Genet..

[7] Ackerman M.J., Priori S.G., Willems S., Berul C., Brugada R., Calkins H., Camm A.J., Ellinor P.T., Gollob M., Hamilton R. (2011). HRS/EHRA expert consensus statement on the state of genetic testing for the channelopathies and cardiomyopathies this document was developed as a partnership between the Heart Rhythm Society (HRS) and the European Heart Rhythm Association (EHRA). Heart Rhythm.

[8] Curran M.E., Splawski I., Timothy K.W., Vincent G.M., Green E.D., Keating M.T. (1995). A molecular basis for cardiac arrhythmia: HERG mutations cause long QT syndrome. Cell.

[9] Sanguinetti M.C., Jiang C., Curran M.E., Keating M.T. (1995). A mechanistic link between an inherited and an acquired cardiac arrhythmia: HERG encodes the IKr potassium channel. Cell.

[10] Ono M., Burgess D.E., Schroder E.A., Elayi C.S., Anderson C.L., January C.T., Sun B., Immadisetty K., Kekenes-Huskey P.M., Delisle B.P. (2020). Long QT Syndrome Type 2: Emerging Strategies for Correcting Class 2 KCNH2 (hERG) Mutations and Identifying New Patients. Biomolecules.

[11] Lahrouchi N., Tadros R., Crotti L., Mizusawa Y., Postema P.G., Beekman L., Walsh R., Hasegawa K., Barc J., Ernsting M. (2020). Transethnic Genome-Wide Association Study Provides Insights in the Genetic Architecture and Heritability of Long QT Syndrome. Circulation.

[12] Priori S.G., Napolitano C., Schwartz P.J. (1999). Low penetrance in the long-QT syndrome: Clinical impact. Circulation.

[13] Smulyan H. (2018). QT interval: Bazett’s Correction corrected. J. Electrocardiol..

[14] Bazett H.C. (1920). An analysis of the time-relations of the electrocardiogram. Heart.

[15] Miller S.A., Dykes D.D., Polesky H.F. (1988). A simple salting out procedure for extracting DNA from human nucleated cells. Nucleic Acids Res..

[16] Gómez J., Reguero J.R., Morís C., Martín M., Alvarez V., Alonso B., Iglesias S., Coto E. (2014). Mutation analysis of the main hypertrophic cardiomyopathy genes using multiplex amplification and semiconductor next-generation sequencing. Circ. J. Off. J. Jpn. Circ. Soc..

[17] Gómez J., Lorca R., Reguero J.R., Morís C., Martín M., Tranche S., Alonso B., Iglesias S., Alvarez V., Díaz-Molina B. (2017). Screening of the Filamin C Gene in a Large Cohort of Hypertrophic Cardiomyopathy Patients. Circ. Cardiovasc. Genet..

[18] Richards S., Aziz N., Bale S., Bick D., Das S., Gastier-Foster J., Grody W.W., Hegde M., Lyon E., Spector E. (2015). Standards and guidelines for the interpretation of sequence variants: A joint consensus recommendation of the American College of Medical Genetics and Genomics and the Association for Molecular Pathology. Genet. Med. Off. J. Am. Coll. Med. Genet..

[19] Gnecchi M., Sala L., Schwartz P.J. (2021). Precision Medicine and cardiac channelopathies: When dreams meet reality. Eur. Heart J..

[20] Smith J.L., Anderson C.L., Burgess D.E., Elayi C.S., January C.T., Delisle B.P. (2016). Molecular pathogenesis of long QT syndrome type 2. J. Arrhythmia.

[21] Gong Q., Zhang L., Vincent G.M., Horne B.D., Zhou Z. (2007). Nonsense mutations in hERG cause a decrease in mutant mRNA transcripts by nonsense-mediated mRNA decay in human long-QT syndrome. Circulation.

[22] Locati E.T. (2006). QT interval duration remains a major risk factor in long QT syndrome patients. J. Am. Coll. Cardiol..

[23] Schwartz P.J., Priori S.G., Spazzolini C., Moss A.J., Vincent G.M., Napolitano C., Denjoy I., Guicheney P., Breithardt G., Keating M.T. (2001). Genotype-phenotype correlation in the long-QT syndrome: Gene-specific triggers for life-threatening arrhythmias. Circulation.

[24] Al-Khatib S.M., Stevenson W.G., Ackerman M.J., Bryant W.J., Callans D.J., Curtis A.B., Deal B.J., Dickfeld T., Field M.E., Fonarow G.C. (2018). 2017 AHA/ACC/HRS guideline for management of patients with ventricular arrhythmias and the prevention of sudden cardiac death: Executive summary: A Report of the American College of Cardiology/American Heart Association Task Force on Clinical Practice Guidelines and the Heart Rhythm Society. Heart Rhythm.

[25] Schwartz P.J., Crotti L., Insolia R. (2012). Long-QT syndrome: From genetics to management. Circ. Arrhythm. Electrophysiol..

[26] Shah M.J., Silka M.J., Silva J.N.A., Balaji S., Beach C.M., Benjamin M.N., Berul C.I., Cannon B., Cecchin F., Writing Committee Members (2021). 2021 PACES Expert Consensus Statement on the Indications and Management of Cardiovascular Implantable Electronic Devices in Pediatric Patients. Heart Rhythm.

[27] Priori S.G., Blomström-Lundqvist C., Mazzanti A., Blom N., Borggrefe M., Camm J., Elliott P.M., Fitzsimons D., Hatala R., Hindricks G. (2015). 2015 ESC Guidelines for the management of patients with ventricular arrhythmias and the prevention of sudden cardiac death: The Task Force for the Management of Patients with Ventricular Arrhythmias and the Prevention of Sudden Cardiac Death of the European Society of Cardiology (ESC). Endorsed by: Association for European Paediatric and Congenital Cardiology (AEPC). Eur. Heart J..

[28] Shimizu W., Moss A.J., Wilde A.A.M., Towbin J.A., Ackerman M.J., January C.T., Tester D.J., Zareba W., Robinson J.L., Qi M. (2009). Genotype-phenotype aspects of type 2 long QT syndrome. J. Am. Coll. Cardiol..

[29] Moss A.J., Zareba W., Kaufman E.S., Gartman E., Peterson D.R., Benhorin J., Towbin J.A., Keating M.T., Priori S.G., Schwartz P.J. (2002). Increased risk of arrhythmic events in long-QT syndrome with mutations in the pore region of the human ether-a-go-go-related gene potassium channel. Circulation.

[30] Tester D.J., Will M.L., Haglund C.M., Ackerman M.J. (2005). Compendium of cardiac channel mutations in 541 consecutive unrelated patients referred for long QT syndrome genetic testing. Heart Rhythm.

[31] Splawski I., Shen J., Timothy K.W., Lehmann M.H., Priori S., Robinson J.L., Moss A.J., Schwartz P.J., Towbin J.A., Vincent G.M. (2000). Spectrum of mutations in long-QT syndrome genes. KVLQT1, HERG, SCN5A, KCNE1, and KCNE2. Circulation.

[32] Kapa S., Tester D.J., Salisbury B.A., Harris-Kerr C., Pungliya M.S., Alders M., Wilde A.A.M., Ackerman M.J. (2009). Genetic testing for long-QT syndrome: Distinguishing pathogenic mutations from benign variants. Circulation.

[33] Rashba E.J., Zareba W., Moss A.J., Hall W.J., Robinson J., Locati E.H., Schwartz P.J., Andrews M. (1998). Influence of pregnancy on the risk for cardiac events in patients with hereditary long QT syndrome. LQTS Investigators. Circulation.

[34] Seth R., Moss A.J., McNitt S., Zareba W., Andrews M.L., Qi M., Robinson J.L., Goldenberg I., Ackerman M.J., Benhorin J. (2007). Long QT syndrome and pregnancy. J. Am. Coll. Cardiol..

[35] Ishibashi K., Aiba T., Kamiya C., Miyazaki A., Sakaguchi H., Wada M., Nakajima I., Miyamoto K., Okamura H., Noda T. (2017). Arrhythmia risk and β-blocker therapy in pregnant women with long QT syndrome. Heart Br. Card. Soc..

[36] Garg L., Garg J., Krishnamoorthy P., Ahnert A., Shah N., Dusaj R.S., Bozorgnia B. (2017). Influence of Pregnancy in Patients With Congenital Long QT Syndrome. Cardiol. Rev..

[37] Goldenberg I., Bradley J., Moss A., McNitt S., Polonsky S., Robinson J.L., Andrews M., Zareba W. (2010). International LQTS Registry Investigators Beta-blocker efficacy in high-risk patients with the congenital long-QT syndrome types 1 and 2: Implications for patient management. J. Cardiovasc. Electrophysiol..

[38] Schwartz P.J., Priori S.G., Locati E.H., Napolitano C., Cantù F., Towbin J.A., Keating M.T., Hammoude H., Brown A.M., Chen L.S. (1995). Long QT syndrome patients with mutations of the SCN5A and HERG genes have differential responses to Na+ channel blockade and to increases in heart rate. Implications for gene-specific therapy. Circulation.

[39] Bos J.M., Crotti L., Rohatgi R.K., Castelletti S., Dagradi F., Schwartz P.J., Ackerman M.J. (2019). Mexiletine Shortens the QT Interval in Patients With Potassium Channel-Mediated Type 2 Long QT Syndrome. Circ. Arrhythm. Electrophysiol..

[40] Dusi V., Pugliese L., De F.G.M., Odero A., Crotti L., Dagradi F., Castelletti S., Vicentini A., Rordorf R., Li C. (2022). Left Cardiac Sympathetic Denervation for Long QT Syndrome. JACC Clin. Electrophysiol..

[41] Schwartz P.J., Priori S.G., Cerrone M., Spazzolini C., Odero A., Napolitano C., Bloise R., De Ferrari G.M., Klersy C., Moss A.J. (2004). Left cardiac sympathetic denervation in the management of high-risk patients affected by the long-QT syndrome. Circulation.

[42] Schwartz P.J., Spazzolini C., Priori S.G., Crotti L., Vicentini A., Landolina M., Gasparini M., Wilde A.A.M., Knops R.E., Denjoy I. (2010). Who are the long-QT syndrome patients who receive an implantable cardioverter-defibrillator and what happens to them? Data from the European Long-QT Syndrome Implantable Cardioverter-Defibrillator (LQTS ICD) Registry. Circulation.

