# Supplementary material for: KCNH2 p.Gly262AlafsTer98: A New Threatening Variant Associated with Long QT Syndrome in a Spanish Cohort
Source: Life (Basel). 2022 Apr 8;12(4):556. doi: 10.3390/life12040556 (PMC9024605; doi:10.3390/life12040556)
Supplement: Supplementary file 1 [file life-12-00556-s001.zip › life-1619808-supplementary.pdf]

life

MDPI

Figure S1: Sanger electropherogram of *KCNH2* exon 4 PCR fragment displaying p.(Gly262AlafsTer98) variant.

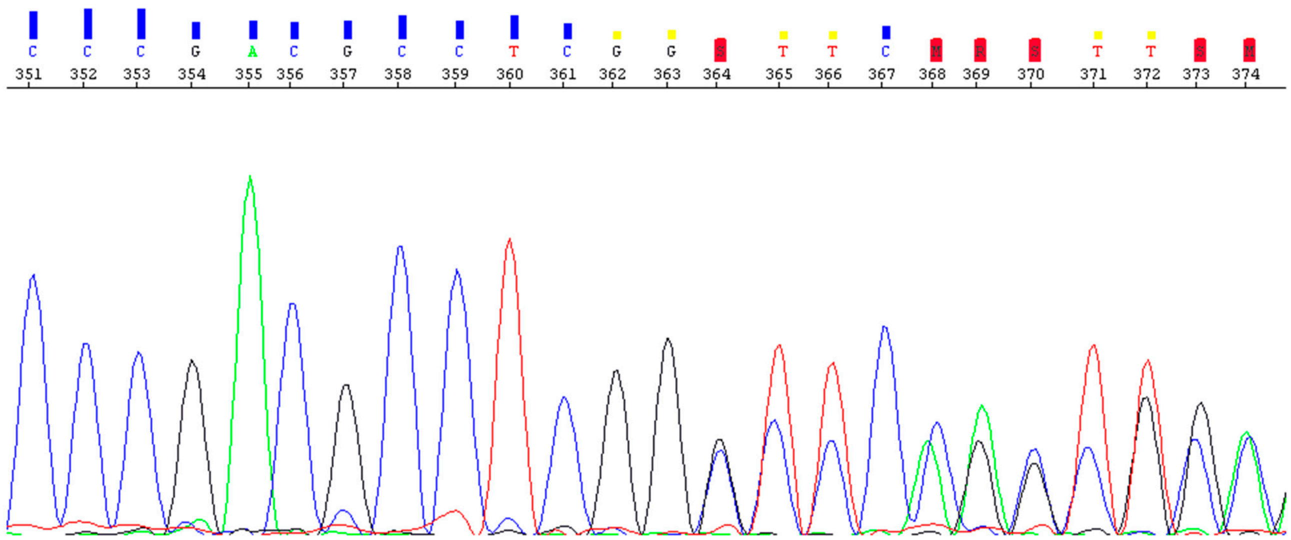

Table S1. Genes included in the NGS, including LQTS-associated genes.

Gene	Chr	Num Amplicons	Total Bases	Covered Bases	Missed Bases	Overall Coverage (parts per unit)
ABCC8	chr11	47	5139	5139	0	1
ABCC9	chr12	44	5178	5178	0	1
ACTA2	chr10	8	1214	1214	0	1
ACTC1	chr15	8	1194	1194	0	1
ACTN2	chr1	28	3112	3112	0	1
ACVRL1	chr12	14	1602	1602	0	1
ADAMTSL4	chr1	31	4269	4269	0	1
AGL	chr1	52	6024	6024	0	1
AKAP9	chr7	90	12224	12224	0	1
ALMS1	chr2	85	12734	12734	0	1
ANK2	chr4	93	12411	12411	0	1
ANKRD1	chr10	9	1050	1050	0	1
APOB	chr2	85	13982	13982	0	1
ATP7A	chrX	41	4723	4723	0	1
B3GAT3	chr11	13	1474	1471	3	0.998
BAG3	chr10	12	1768	1768	0	1
BGN	chrX	12	1457	1457	0	1
BLK	chr8	21	2118	2118	0	1
BMP10	chr2	9	1375	1375	0	1
BMPR1A	chr10	13	1709	1709	0	1
BMPR1B	chr4	13	1709	1709	0	1
BMPR2	chr2	25	3247	3247	0	1

BRAF	chr7	23	2481	2481	0	1
CACNA1C	chr12	64	7626	7626	0	1
CACNA1D	chr3	64	7150	7150	0	1
CACNA2D1	chr7	51	5327	5327	0	1
CACNB2	chr10	26	3269	3269	0	1
CALM1	chr14	7	690	690	0	1
CALM2	chr2	7	664	664	0	1
CALM3	chr19	8	750	750	0	1
CALR3	chr19	14	1605	1574	31	0.9807
CASQ2	chr1	13	1750	1750	0	1
CAV3	chr3	4	476	476	0	1
CBL	chr11	23	2881	2881	0	1
CBS	chr21	21	1806	1806	0	1
CHRM2	chr7	8	1451	1451	0	1
COL1A1	chr17	56	4905	4905	0	1
COL1A2	chr7	55	4621	4621	0	1
COL3A1	chr2	57	4911	4911	0	1
COL5A1	chr9	78	6256	6256	0	1
COL5A2	chr2	64	7200	7200	0	1
CRYAB	chr11	5	678	678	0	1
CSRP3	chr11	6	635	635	0	1
CTNNA3	chr10	29	3758	3758	0	1
CTNNB1	chr3	19	2486	2486	0	1
DES	chr2	15	1503	1503	0	1
DMD	chrX	106	12161	12161	0	1
DNAJC19	chr3	6	411	411	0	1
DOLK	chr9	9	1667	1667	0	1
DSC2	chr18	25	2912	2912	0	1
DSG2	chr18	28	3507	3507	0	1
DSP	chr6	57	8856	8856	0	1
DTNA	chr18	29	3795	3795	0	1
EIF2AK3	chr2	32	4201	4201	0	1
EIF2AK4	chr15	51	6900	6900	0	1
ELN	chr7	37	4094	4094	0	1
EMD	chrX	9	1005	1005	0	1
ENG	chr9	22	2753	2753	0	1
EYA4	chr6	26	3039	3039	0	1
FBN1	chr15	73	9266	9266	0	1
FBN2	chr5	88	9389	9389	0	1
FHL1	chrX	11	1472	1466	6	0.9959
FHL2	chr2	9	1090	1090	0	1
FKRP	chr19	11	1498	1498	0	1
FKTN	chr9	13	1509	1509	0	1
FLNA	chrX	76	10294	10294	0	1
FLNC	chr7	75	8658	8658	0	1

GAA	chr17	27	3049	3049	0	1
GATA4	chr8	12	1392	1392	0	1
GATA5	chr20	12	1494	1494	0	1
GATAD1	chr7	9	1060	1060	0	1
GCK	chr7	17	1608	1608	0	1
GDF2	chr10	7	1390	1390	0	1
GJA1	chr6	6	1159	1159	0	1
GJA5	chr1	8	1117	1117	0	1
GLA	chrX	10	1360	1360	0	1
GLIS3	chr9	22	3293	3293	0	1
GPD1L	chr3	11	1376	1376	0	1
HFE	chr6	9	1125	1107	18	0.984
HNF1A	chr12	16	2017	2017	0	1
HNF1B	chr17	16	1856	1856	0	1
HNF4A	chr20	20	1860	1860	0	1
HRAS	chr11	5	683	683	0	1
ILK	chr11	14	1959	1959	0	1
JPH2	chr20	17	2402	2402	0	1
JUP	chr17	21	2368	2368	0	1
KCNA5	chr12	11	1892	1892	0	1
KCND2	chr7	15	2193	2193	0	1
KCND3	chr1	17	2318	2318	0	1
KCNE1	chr21	2	400	400	0	1
KCNE2	chr21	2	382	382	0	1
KCNE3	chr11	2	362	362	0	1
KCNE5	chrX	3	479	479	0	1
KCNH2	chr7	28	4017	4017	0	1
KCNJ11	chr11	6	1183	1183	0	1
KCNJ2	chr17	8	1294	1294	0	1
KCNJ5	chr11	9	1340	1340	0	1
KCNJ8	chr12	8	1375	1375	0	1
KCNK3	chr2	9	1285	1285	0	1
KCNQ1	chr11	20	2206	2206	0	1
KLF11	chr2	11	1739	1739	0	1
KRAS	chr12	6	737	737	0	1
LAMA4	chr6	55	7540	7540	0	1
LAMP2	chrX	15	1626	1626	0	1
LDB3	chr10	24	2668	2664	4	0.9985
LDLR	chr19	21	2763	2763	0	1
LDLRAP1	chr1	14	1377	1377	0	1
LIPA	chr10	14	1650	1650	0	1
LMNA	chr1	18	2369	2369	0	1
LOX	chr5	13	1604	1604	0	1
LZTR1	chr22	29	2733	2733	0	1
MAP2K1	chr15	12	1292	1292	0	1

MAP2K2	chr19	13	1313	1313	0	1
MAT2A	chr2	11	1638	1638	0	1
MED12	chrX	59	6984	6984	0	1
MFAP5	chr12	9	972	972	0	1
MIB1	chr18	36	4071	4071	0	1
MURC	chr9	7	1195	1195	0	1
MYBPC3	chr11	35	4155	4155	0	1
MYH11	chr16	51	6391	6391	0	1
MYH6	chr14	51	6190	6190	0	1
MYH7	chr14	45	6188	6188	0	1
MYL2	chr12	7	571	571	0	1
MYL3	chr3	6	648	648	0	1
MYLK	chr3	41	6055	6055	0	1
MYLK2	chr20	21	2391	2391	0	1
MYOM1	chr18	56	6908	6908	0	1
MYOZ2	chr4	8	1045	1045	0	1
MYPN	chr10	36	4983	4983	0	1
NEBL	chr10	38	5002	5002	0	1
NEUROD1	chr2	6	1121	1121	0	1
NEUROG3	chr10	5	695	695	0	1
NEXN	chr1	21	2628	2628	0	1
NF1	chr17	74	9161	9161	0	1
NKX2-5	chr5	9	1142	1142	0	1
NKX2-6	chr8	6	926	926	0	1
NNT	chr5	37	4311	4306	5	0.9988
NOTCH1	chr9	63	8008	7987	21	0.9974
NOTCH3	chr19	51	7296	7296	0	1
NPPA	chr1	4	486	486	0	1
NRAS	chr1	5	610	610	0	1
PAX6	chr11	13	2035	2035	0	1
PCSK9	chr1	16	2199	2199	0	1
PDLIM3	chr4	13	1733	1733	0	1
PDX1	chr13	7	952	952	0	1
PITX2	chr4	11	1209	1209	0	1
PKP2	chr12	21	2786	2786	0	1
PLN	chr6	1	169	169	0	1
PLOD1	chr1	28	3325	3325	0	1
PPARG	chr3	12	1588	1588	0	1
PRDM16	chr1	37	4681	4654	27	0.9942
PRKAG2	chr7	19	1974	1974	0	1
PRKG1	chr10	26	3087	3087	0	1
PSEN1	chr14	12	1504	1504	0	1
PSEN2	chr1	15	1447	1447	0	1
PTPN11	chr12	16	1936	1936	0	1
RAF1	chr3	18	2107	2107	0	1

RANGRF	chr17	8	853	853	0	1
RASA1	chr5	39	3412	3411	1	0.9997
RASA2	chr3	38	3815	3793	22	0.9942
RBM20	chr10	27	3824	3817	7	0.9982
RFX6	chr6	30	3737	3737	0	1
RIT1	chr1	8	771	771	0	1
RYR2	chr1	134	15954	15954	0	1
SCN10A	chr3	49	7221	7221	0	1
SCN1B	chr19	9	1066	1066	0	1
SCN2B	chr11	7	848	848	0	1
SCN3B	chr11	8	898	898	0	1
SCN4B	chr11	7	887	887	0	1
SCN5A	chr3	44	6423	6423	0	1
SDHA	chr5	19	2145	2145	0	1
SGCD	chr5	8	1025	1025	0	1
SHOC2	chr10	14	1829	1829	0	1
SLC19A2	chr1	13	1794	1794	0	1
SLC22A5	chr5	14	1856	1856	0	1
SLC2A10	chr20	14	1876	1876	0	1
SLMAP	chr3	33	3799	3799	0	1
SMAD1	chr4	12	1698	1698	0	1
SMAD2	chr18	13	1504	1504	0	1
SMAD3	chr15	14	1452	1452	0	1
SMAD4	chr18	18	1769	1769	0	1
SMAD9	chr13	13	1704	1704	0	1
SNTA1	chr20	17	1918	1918	0	1
SOS1	chr2	40	4232	4232	0	1
SOS2	chr14	46	4919	4919	0	1
SPRED1	chr15	13	1405	1405	0	1
STAP1	chr4	10	1248	1248	0	1
TAZ	chrX	14	1043	1043	0	1
TBX5	chr12	12	1637	1637	0	1
TCAP	chr17	5	524	524	0	1
TGFB2	chr1	12	1409	1409	0	1
TGFB3	chr14	10	1589	1589	0	1
TGFBR1	chr9	13	1614	1614	0	1
TGFBR2	chr3	12	1859	1859	0	1
TMEM43	chr3	13	1323	1323	0	1
TMPO	chr12	25	3385	3385	0	1
TNNC1	chr3	7	546	546	0	1
TNNI3	chr19	7	702	702	0	1
TNNI3K	chr1	30	3758	3710	48	0.9872
TNNT2	chr1	17	1081	1081	0	1
TOPBP1	chr3	51	5919	5919	0	1
TPM1	chr15	15	1500	1500	0	1

TRDN	chr6	48	4418	4418	0	1
TRPM4	chr19	32	3895	3895	0	1
TTN	chr2	800	132581	129775	2806	0.9788
TTR	chr18	5	484	484	0	1
TXNRD2	chr22	23	2543	2543	0	1
VCL	chr10	36	4505	4505	0	1
WFS1	chr4	19	2743	2743	0	1
ZDHHC9	chrX	10	1545	1545	0	1
